# AGE DIFFERENCES IN POSTPRANDIAL AMINO ACID PROFILES ARE DETERMINED BY PROTEIN CONTENT OF THE MEAL

**DOI:** 10.1093/geroni/igae098.2830

**Published:** 2024-12-31

**Authors:** Catrin Herpich, Donna Li, Daniela Weber, Kristina Norman

**Affiliations:** German Institute of Human Nutrition Potsdam-Rehbrücke, Nuthetal, Brandenburg, Germany; German Institute of Human Nutrition Potsdam-Rehbrücke, Nuthetal, Brandenburg, Germany; German Institute of Human Nutrition Potsdam-Rehbrücke, Nuthetal, Brandenburg, Germany; German Institute of Human Nutrition Potsdam-Rehbrücke, Nuthetal, Brandenburg, Germany

## Abstract

Amino acid availability after a meal differs between older and younger adults and the macronutrient composition of the meal affects postprandial amino acid availability as well. In this analysis, we examined postprandial amino acid response of healthy older and younger study participants to test meals with varying protein contents. The test meal either had normal protein (16 E% (energy percent) protein, 68 E% carbohydrates) or a high-protein content (HP, 77 E% protein (whey), 17% carbohydrates; 450 kcal each). Postprandial plasma amino acid concentrations were quantified at different timepoints (0, 30, 60, 120, 240 min) using UPLC-MS/MS. Amino acids were grouped into essential (EAA), dispensable (NEAA), and branched-chain amino acids (BCAA). Time and group differences of amino acid profiles were examined by repeated measures ANOVA and by comparing the incremental area under the curve (iAUC) using Mann-Whitney test. In older (n=40, 72.8±5.05 years) and younger adults (n=30, 25.5±3.94 years), EAA, NEAA and BCAA concentrations increased after both test meals over four hours (all p< 0.005), with a higher increase in response to HP. After the HP meal, significantly higher BCAA and EAA concentrations were found in older adults at 60 min. In addition, iAUC of BCAA, EAA and NEAA was significantly higher only in older participants following HP (all p< 0.05). After a meal containing high amounts of fast-absorbed protein, healthy older adults show higher postprandial amino acid concentrations that might result from delayed tissue uptake. Whereas in response to normal protein content no age-differences were seen.

